# The Diurnal Logic of the Expression of the Chloroplast Genome in *Chlamydomonas reinhardtii*

**DOI:** 10.1371/journal.pone.0108760

**Published:** 2014-10-01

**Authors:** Adam D. Idoine, Alix Boulouis, Jens Rupprecht, Ralph Bock

**Affiliations:** Max-Planck-Institut für Molekulare Pflanzenphysiologie, Potsdam, Golm, Germany; Leibniz-Institute for Vegetable and Ornamental Plants, Germany

## Abstract

Chloroplasts are derived from cyanobacteria and have retained a bacterial-type genome and gene expression machinery. The chloroplast genome encodes many of the core components of the photosynthetic apparatus in the thylakoid membranes. To avoid photooxidative damage and production of harmful reactive oxygen species (ROS) by incompletely assembled thylakoid protein complexes, chloroplast gene expression must be tightly regulated and co-ordinated with gene expression in the nucleus. Little is known about the control of chloroplast gene expression at the genome-wide level in response to internal rhythms and external cues. To obtain a comprehensive picture of organelle transcript levels in the unicellular model alga *Chlamydomonas reinhardtii* in diurnal conditions, a qRT-PCR platform was developed and used to quantify 68 chloroplast, 21 mitochondrial as well as 71 nuclear transcripts in cells grown in highly controlled 12 h light/12 h dark cycles. Interestingly, in anticipation of dusk, chloroplast transcripts from genes involved in transcription reached peak levels first, followed by transcripts from genes involved in translation, and finally photosynthesis gene transcripts. This pattern matches perfectly the theoretical demands of a cell “waking up” from the night. A similar trend was observed in the nuclear transcripts. These results suggest a striking internal logic in the expression of the chloroplast genome and a previously unappreciated complexity in the regulation of chloroplast genes.

## Introduction

The unicellular green alga *Chlamydomonas reinhardtii* has become a model organism for many research areas, including chloroplast biology and photosynthesis research [1], [2], [3], [4], [5]. Moreover, its capacity to produce molecular hydrogen and to synthesize and accumulate considerable amounts of oil also have made *C. reinhardtii* an attractive target organism of many efforts to develop algae as an efficient and sustainable production platform for biofuels [6], [7], [8], [9]. Since all algal biofuels and renewable green chemical feedstocks are directly or indirectly derived from photosynthesis and many of the core components of the photosynthetic apparatus are encoded in the chloroplast (plastid) DNA, a comprehensive understanding of the mechanisms that govern expression of the chloroplast genome is of great importance to all future attempts to rationally engineer primary metabolism. However, our knowledge about the regulatory mechanisms operating in the control of chloroplast gene expression in response to endogenous cues and environmental stimuli and the signaling pathways co-ordinating expression of the chloroplast genome with that of the nuclear genome is still far from complete.

The chloroplast genome of *C. reinhardtii* is 204 kb in size and contains 99 genes [10]. The vast majority of these genes belong to the two main gene classes found in plastid genomes of algae and embryophyte plants: photosynthesis genes and genetic system genes [11]. The photosynthesis-related genes comprise genes for subunits of the major multiprotein complexes in the thylakoid membrane that participate in photosynthetic electron transfer and ATP generation (photosystem II, cytochrome b_6_f complex, photosystem I, ATP synthase). The genetic system genes encode components of the transcriptional apparatus (subunits of the plastid-encoded RNA polymerase, PEP) and the translational machinery (rRNAs, tRNAs, ribosomal proteins).

Genes in the chloroplast genome of *C. reinhardtii* are transcribed by a multisubunit RNA polymerase that resembles bacterial RNA polymerases of the α_2_ββ’ type. All of its core subunits are encoded in the chloroplast DNA and, for this reason, the enzyme has been dubbed PEP (for plastid-encoded RNA polymerase). In *Chlamydomonas*, there appears to be no nucleus-encoded plastid RNA polymerase that has been shown to be additionally present in plastids of embryophyte plants [12], [13]. The PEP core complex interacts with the single sigma factor found in the nuclear genome of *C. reinhardtii* (RPOD) to confer sequence specificity in promoter recognition [14], [15]. TATA-like -10 box sequences (and, in some cases, the extended -10 box motif TATAATAT) are found upstream of many reading frames in the *C. reinhardtii* plastid DNA and are thought to act as key promoter elements. However, several other types of promoters have been identified, and a consensus is yet to emerge [16], [17], [18].

Natural conditions for *C. reinhardtii* involve several environmental variables which vary reliably over a day. The strongest signal over a day is light, which is often used in laboratory conditions to simulate a diurnal cycle. Many cellular processes respond to light-dark cycles, such as regulation of the cell cycle, as well as various steps of chloroplast gene expression. As examples, the structure of the entire plastid genome is most accessible early in the light period, and the half-life of several mRNAs is much shorter in the light period than in the dark [19], [20].

So far, nine chloroplast transcripts have been quantified over a diurnal cycle in at least one study (Figure S1 in File S1). The majority of these attain their highest levels in the light period. However, the majority of the mRNAs which have been studied so far are involved in photosynthesis. Neither RNA polymerase subunits nor any subunits of the ribosome have been analyzed. Interestingly, when the diurnal regulation of chloroplast transcription in *C. reinhardtii* was studied, it was found to be accompanied by little or no fluctuation in RNA polymerase activity or in the level of RPOD, the only sigma factor in chloroplasts of *Chlamydomonas* [21].

Here, we have undertaken a systematic study of the expression of the chloroplast genome in *Chlamydomonas* under diurnal conditions. Our work has uncovered a surprising internal logic in the expression of the chloroplast genome in that transcripts of the transcriptional apparatus peak first, followed by transcripts of the translational machinery, and finally by photosynthesis gene transcripts.

## Results

### Development of a qRT-PCR platform for transcript quantification in *Chlamydomonas*

We have previously designed a microarray for profiling of chloroplast transcripts in the model alga *C. reinhardtii* and used it to determine changes in RNA accumulation in response to changing environmental parameters [22]. While the microarray faithfully detected expression changes in genes responding strongly to environmental cues, its sensitivity and reproducibility was not high enough to accurately determine more subtle changes in transcript levels as they occur, for example, over a diurnal cycle. Since PCR-based transcript profiling assays are potentially more sensitive and less prone to technical variation between experiments [23], we sought to establish a qRT-PCR platform for the quantitative analysis of chloroplast transcripts. To this end, primers were designed for all plastid genes and open reading frames (see Material and Methods; Dataset S1). In addition, the mitochondrial genes and a selected set of nuclear genes were included in the platform (Table S1 in File S1; Dataset S1). Test PCRs confirmed that amplicons of the expected sizes could be generated for most genes and cDNAs derived from their transcripts. Where necessary, several oligonucleotides were tested to optimize the PCRs and prevent amplification of non-specific products. Genes which repeatedly failed quality criteria were excluded from the platform. These included the tRNA genes and several genes encoding small polypeptides, owing to difficulty in designing suitable primers for small sequences. The final design of the fully optimized platform comprises 68 chloroplast, 21 mitochondrial and 51 nuclear transcripts (Table S1 in File S1; Dataset S1).

**Figure 1:**
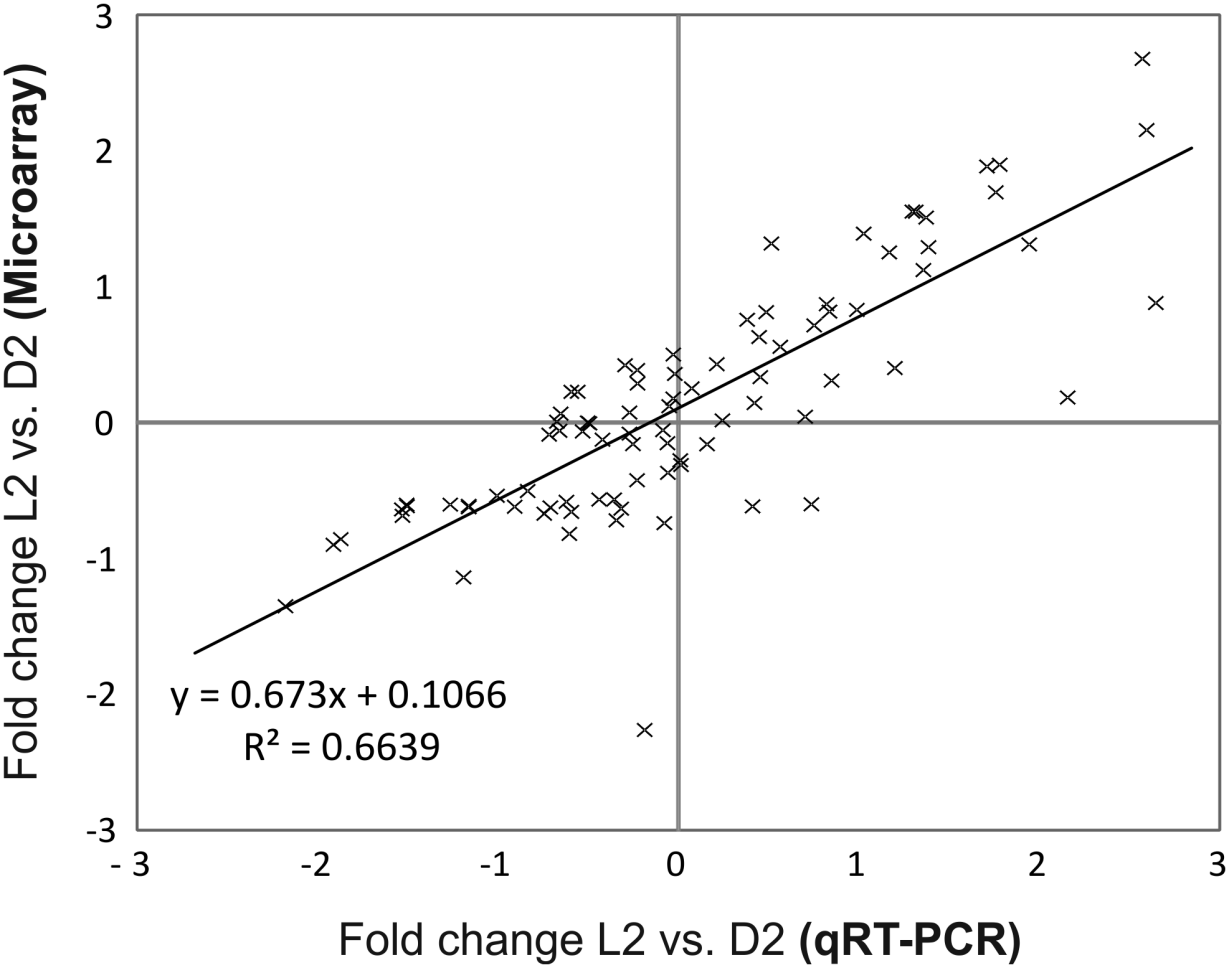
Comparison of microarray and qRT-PCR-based quantification of transcripts in a diurnal rhythm. Changes in organelle (chloroplast and mitochondrial) transcript levels between time points L2 and D2 in a wild-type strain of *C. reinhardtii* (CC-124) were determined by microarray and by qRT-PCR. The resulting fold-changes correlate with an R^2^ value of 0.66. It is lower than 1.0, mainly because the microarray produces less accurate results for lowly expressed genes. All data points represent the average of three independent experiments.

The qRT-PCR platform was then validated against our previously designed microarray for profiling of chloroplast transcripts in *Chlamydomonas* [22]. For this purpose, total RNA was isolated from algal cells grown in diurnal conditions in batch cultures, converted into cDNA by random priming and analyzed by both methods. The fold changes observed between the time points 2 hours into the dark period (D2) and 2 hours into the light period (L2) for each gene agree excellently between the two methods (R^2^ = 0.66; Figure 1). However, overall we found the qRT-PCR platform to produce better results for lowly expressed genes (Figure 1).

To validate the qRT-PCR platform to more diverse studies, we compared the absolute expression levels determined in the light with the data generated by three previous *C. reinhardtii* chloroplast transcriptomic studies. The data obtained in this study were compared to data from two independent microarrays [22], [24] and to data obtained from NanoString technology [25]. Excellent correlations were obtained between this work, one of the microarray studies [22] and the NanoString study [25] with correlation coefficients of 0.82 and 0.60, respectively (Figure S1 in File S1). The data produced in the second microarray study [24] showed a lower correlation coefficient (R^2^ = 0.47), but a similar trend was still visible.

Taken together, these results prove that the qRT-PCR platform developed for this work is of high quality. It, therefore, was used to analyze the accumulation of transcripts in cells grown in fully controlled diurnal conditions.

### Diurnal growth

**Figure 2:**
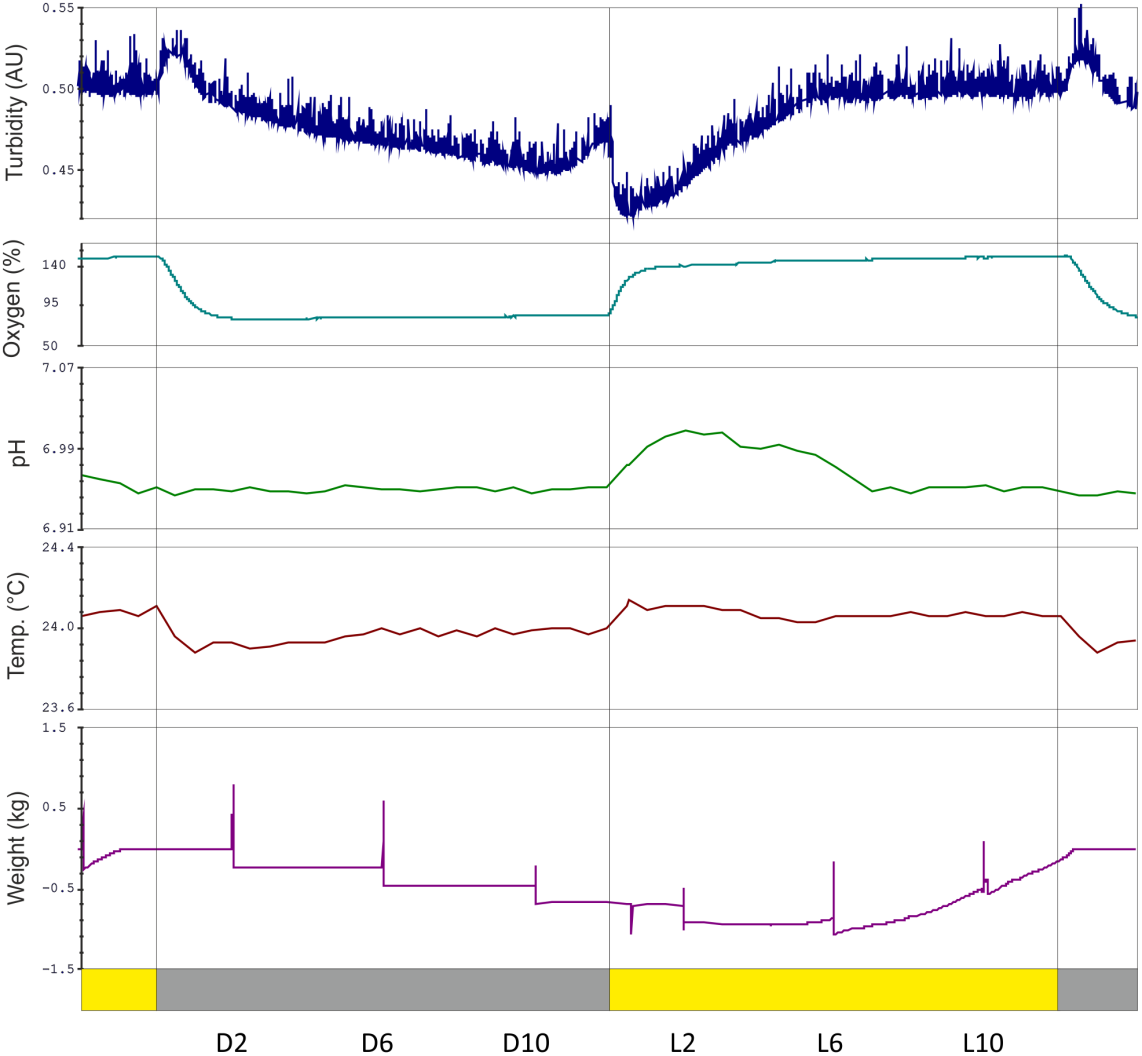
Key bioreactor parameters from a representative bioreactor run (R2) in diurnal conditions. Turbidity (navy blue) slightly decreased during the dark period and increased during the light. The rapid peak after dusk and rapid drop after dawn are technical artefacts of the turbidimeter. The peaks in the weight graph correspond to the sampling times, when the fermenter was disturbed. Dissolved oxygen concentration increases from ca. 80% to ca. 150% very rapidly in the light. The pH of the culture is controlled by titration of acid or base, temperature is controlled by dynamic cooling to maintain reasonably stable values for these parameters. Samples were taken every 4 h, these points are indicated and also visible as peaks in the weight data (purple). Yellow bars represents the 12 h light period (L), flanked by dark periods (D; grey bars).

Cells were grown in highly controlled 5 L bioreactors [22] (see Materials and Methods) which allow very precise control and measurement of culture parameters, such as oxygen concentration, temperature, pH and density of the culture (Figure 2). As expected, the dissolved oxygen concentration in the culture medium increased rapidly at the beginning of the light period to approximately 140 % air saturation due to the rapid onset of photosynthetic oxygen evolution. During the dark period, the dissolved oxygen decreased to around 80 % as the cells consumed some of the dissolved oxygen through respiration, and ceased to generate oxygen from photosynthetic water splitting (Figure 2). Constant bubbling with air ensured that the cells did not experience hypoxia. A slight increase in the medium temperature in the light due to heat produced by the light-emitting diode light shells was also observed. Although the temperature of the bioreactor was dynamically controlled with circulating water, a small increase of 0.15–0.20°C was observed in the light. The pH also fluctuated slightly over the diurnal cycle. It increased at the start of the light period from 6.95 to around 7.0 (Figure 2). This is potentially due to the increase in dissolved oxygen and/or the higher amount of CO_2_ consumed by photosynthesis. The pH then slowly decreased over the rest of the day until a time point between midday (L6) and dusk (L12), when the pH dropped below 6.95. At this point KOH was titrated into the bioreactor to prevent the pH dropping further. KOH titration continued until shortly before midnight (D6), when the pH stabilized and remained at 6.95 until dawn (D12).

The growth of the cells was monitored by the optical density of the culture. The optical density was set to a target value (0.4 or 0.5), but it still showed a complex diurnal pattern. At dawn, a rapid decrease in the optical density was caused directly by light acting on the turbidimeter. The turbidimeter was positioned in the bioreactor to minimize the magnitude of this effect and no changes greater than 0.06 units were observed. The optical density of the culture increased between approximately L1 and L6 as the cells grew, until the target optical density was attained. The culture was then diluted dynamically with fresh medium to maintain a steady state. After the light was turned off, a small peak was observed (again due to a technical effect on the turbidimeter), then the optical density decreased for most of the dark period (presumably due to degradation of intracellular storage compounds). Shortly before the end of the dark period, the optical density began to increase modestly (Figure 2), most likely due to the release of daughter cells.

### Transcript levels over the diurnal cycle determined by qRT-PCR

**Figure 3:**
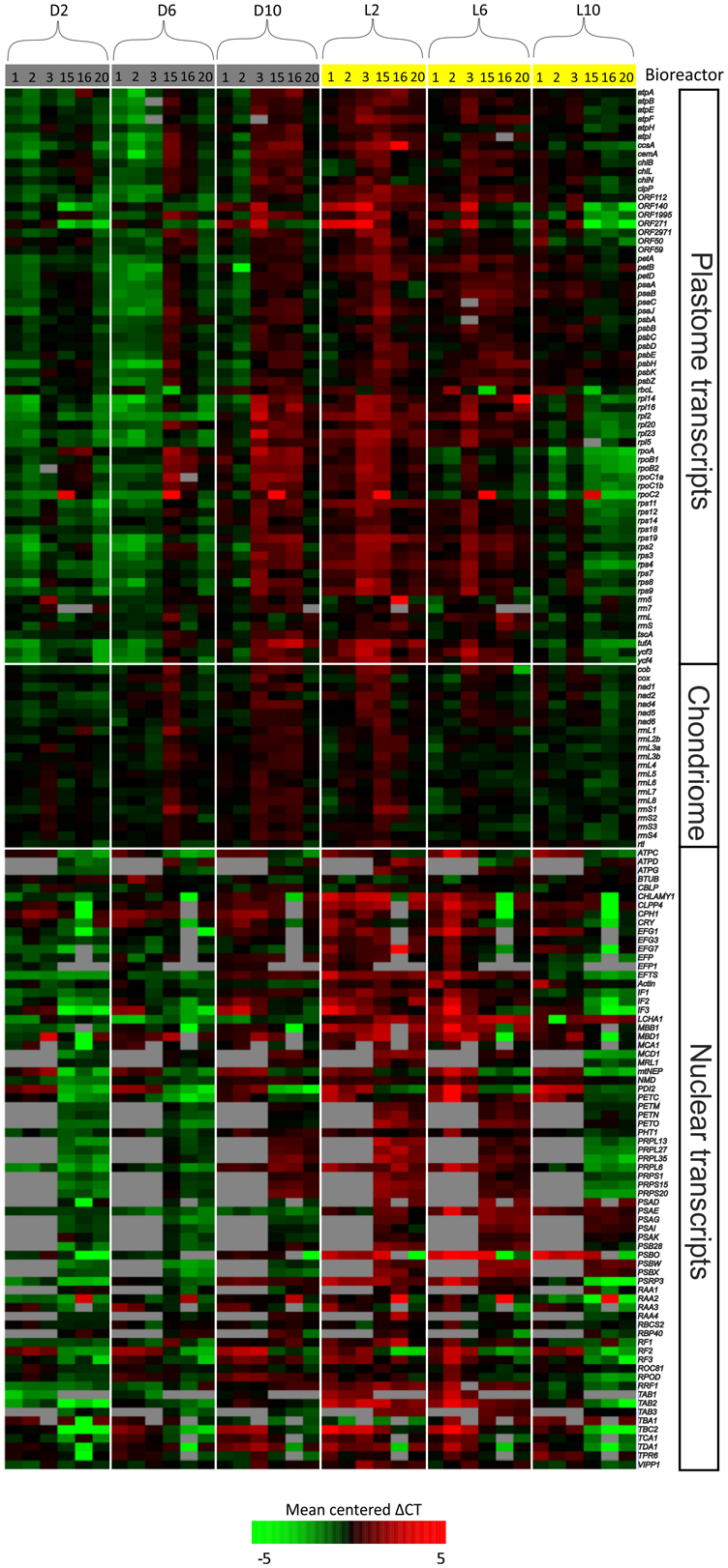
Diurnal transcriptomics on bioreactor cultures of *Chlamydomonas*. The effect of 12 h light/12 h dark cycles on organelle (and selected nuclear) transcripts was analyzed by qRT-PCR. *C. reinhardtii* cells were grown in six independent bioreactor runs (run numbers 1, 2, 3, 15, 16, 20) and harvested at the time points indicated. Data obtained for plastome transcripts is shown in the upper portion of the heatmap, chondriome transcripts in the central region, and nuclear transcripts in the lower region. Within each genome, transcripts are listed alphabetically. Data are normalized to housekeeping transcripts and then to the average across all samples for that gene. Red boxes indicate up-regulation, green boxes: down-regulation, grey boxes: no data. Visualization by Multiexperiment Viewer [38].

**Figure 4:**
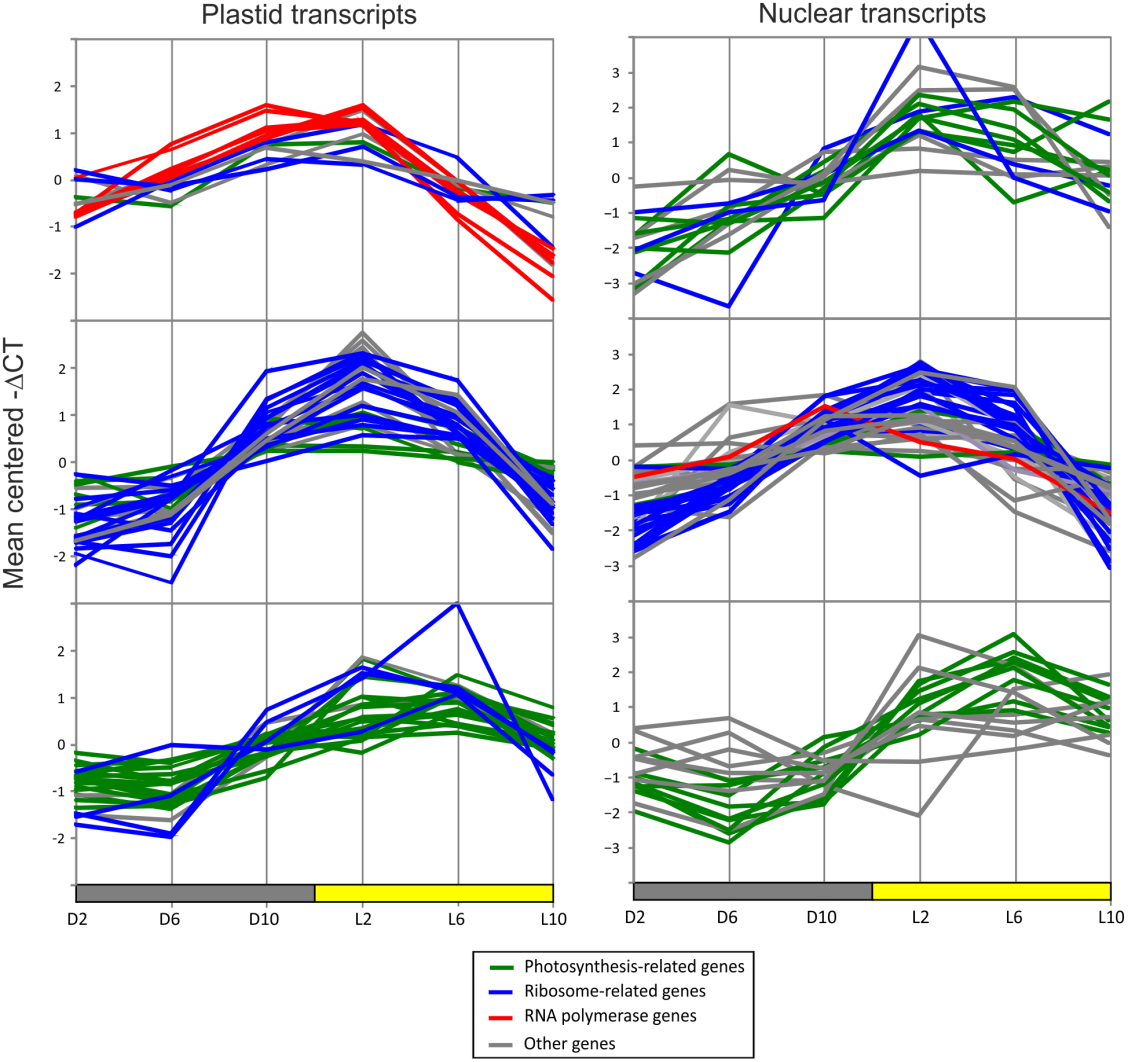
k-means clustering of diurnal qRT-PCR data for chloroplast transcripts (left) and a subset of nuclear transcripts (right). The averaged data for six biological replicates were grouped into three clusters with the Pearson correlation as the distance metric. Gene profiles are colored according to the gene product’s function. Green: photosynthesis-related genes, blue: ribosome-related genes, red: plastid-encoded RNA polymerase genes, grey: miscellaneous genes. Clustering and visualization by Multiexperiment Viewer [38].

RNA was extracted from cells which were sampled from the bioreactors every 4 hours in diurnal conditions. There is a general trend for samples harvested in the light to yield greater amounts of RNA than samples harvested during the dark period (data not shown). To determine changes in transcript levels, the RNA was analyzed by qRT-PCR (Figure 3). Most chloroplast and nuclear transcripts show high levels between at the end of the night and first half of the day (D10 to L6), whereas transcripts derived from the mitochondrial genome (chondriome) do not vary greatly over a diurnal cycle (Figure 3). Although there was some variation between the replicates, the altogether six biological replicates performed allow strong conclusions to be drawn.

k-means clustering was used to group transcripts according to the patterns of their responses to the diurnal conditions. Within the chloroplast transcripts, k-means clustering separates the transcripts into three classes: one containing all PEP transcripts (i.e., mRNAs encoding subunits of the plastid-encoded RNA polymerase), a second one strongly enriched in ribosome-related transcripts, and a third one enriched in photosynthesis-related transcripts (Figure 4). These are the three major classes of protein-coding genes encoded in the *C. reinhardtii* plastid genome (plastome), and the degree of separation is remarkable. Interestingly, a similar separation of photosynthesis- and translation-related transcripts was observed in the subset of nuclear-encoded transcripts which were investigated (Figure 4; Dataset S1), but not in the mitochondrial genes.

**Figure 5:**
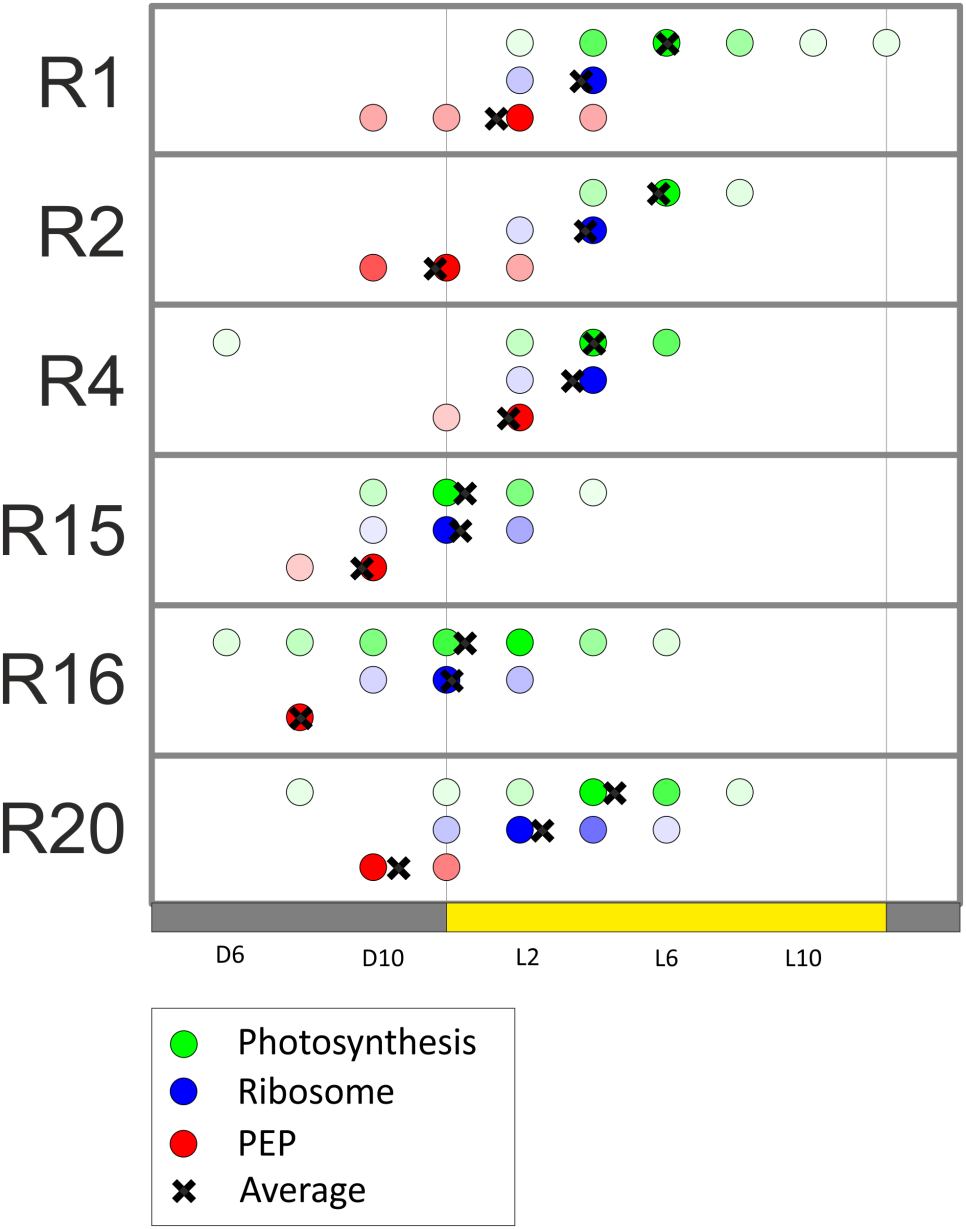
Behavior of functionally related chloroplast transcripts in diurnal conditions. The average peak time of all transcripts belonging to the three functional classes (red: plastid-encoded RNA polymerase genes, PEP; blue: ribosome-related genes; green: photosynthesis-related genes) is shown for each bioreactor experiment. The intensity of the circle represents the number of transcripts peaking at the time, and the black x represents the average of all transcripts. The yellow bar represents the 12 h light period, flanked by dark periods (grey bars).

Figure 4 also shows that most transcripts resemble a sine curve in their diurnal fluctuations. This allowed the quantitation of their phases by comparing each transcript profile to a series of sine curves. The sine curve to which the data for each transcript fitted best, was used to classify the time of the day at which that transcript peaks. This analysis revealed that the defining characteristic of each of the three k-means clusters (Figure 4) is the phase at which the transcripts peak. The PEP cluster peaks first, followed by the ribosome cluster, finally followed by the photosynthesis cluster. Remarkably, in all six experiments, using two different *C. reinhardtii* strains, this order in which the three groups of genes responded remained consistent (Figure 5).

### Northern blot analyses confirm the diurnal patterns in chloroplast transcript levels

**Figure 6:**
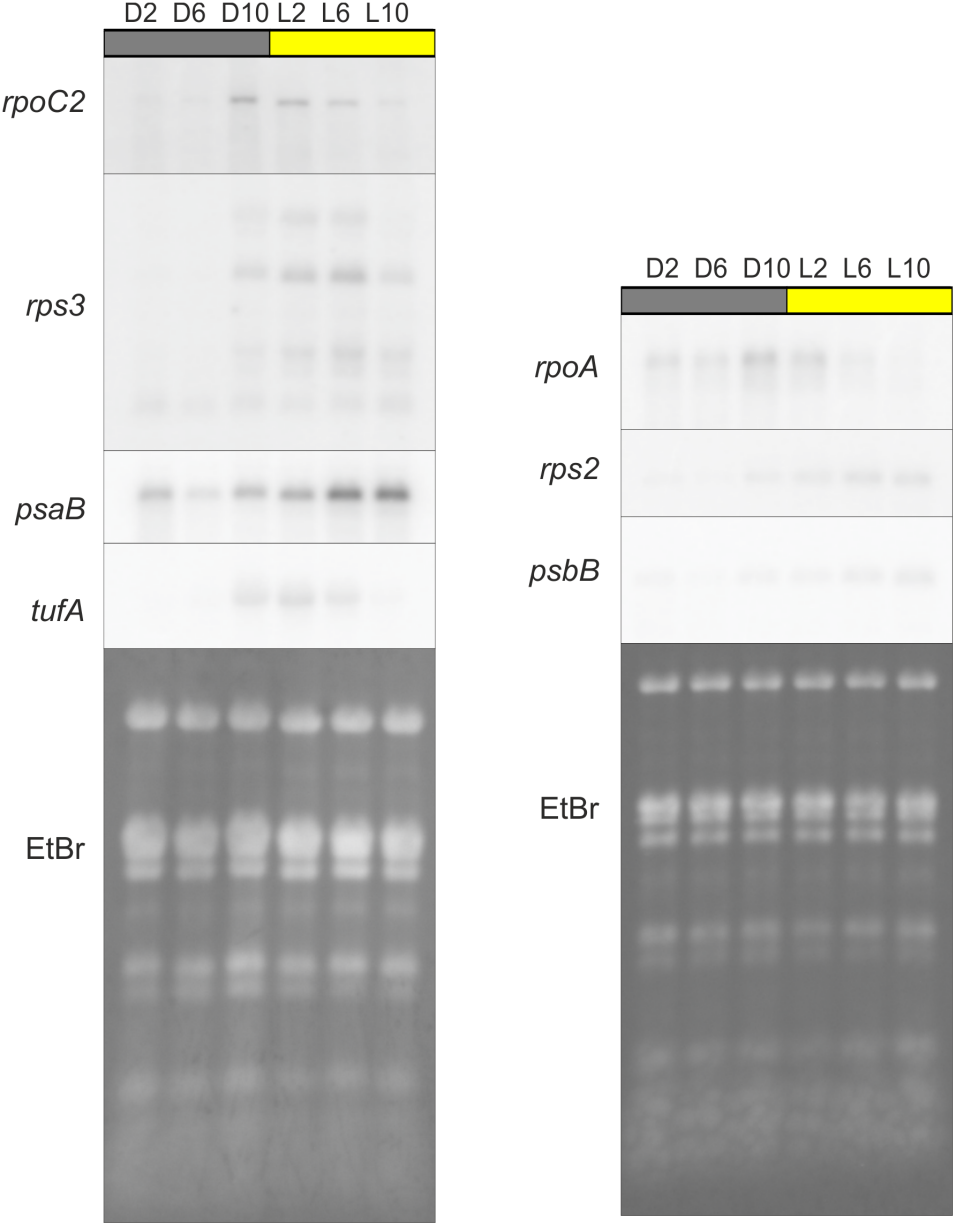
Confirmation of the diurnal expression patterns observed in the pRT-PCR experiments by northern blot analyses. To avoid variation from RNA loading, each membrane was hybridized and stripped multiple times. The order of probing corresponds to the vertical order of the images (and the increasing signal intensity known to be obtained with the different probes). The ethidium bromide fluorescence (EtBr) remaining on the membrane after blotting is shown as a loading control.

In order to confirm the observed striking sequence in diurnal expression of the different classes of chloroplast genes, two representative transcripts from each of the three classes (PEP genes: *rpoA* and *rpoC2*; ribosome-related genes: *rps2* and *rps3*; photosynthesis-related genes: *psaB* and *psbB*) were tested by northern blot analysis (Figure 6). In addition, *tufA*, the gene encoding translation elongation factor EF-Tu [26], [27] was investigated. While being a component of the translational apparatus (and thus belonging to the ribosome-related genes), *tufA* is special in that its expression is known to be under stringent circadian control [28], [29].

The expression profiles determined by northern blot analyses for all seven transcripts agreed excellently with the data obtained from qRT-PCR analysis. *rpoC2* and *rpoA* both reached their highest levels at the end of the night, *rps2* and *rps3* reached their highest levels in the first half of the day, and *psaB* and *psbB* reached high levels in the second half of the day. These results confirm that, during the diurnal cycle, chloroplast transcripts are differentially regulated depending on their function. They also confirm the striking internal logic of chloroplast genome expression (Figures 4–6): the genes encoding the components of the transcriptional machinery are induced and peak shortly before dawn, followed by the genes encoding components of the translational machinery, and finally followed by the genes encoding the components of the photosynthetic apparatus.

## Discussion

In this work, we have used the model alga *C. reinhardtii* to study the expression of the chloroplast and mitochondrial genomes in comparison to a subset of nuclear genes over the diurnal cycle. The qRT-PCR platform developed for this purpose provides a useful tool for systematic analyses of organellar gene expression in *Chlamydomonas* in response to environmental conditions and developmental cues. With respect to sensitivity and reproducibility, the platform compares favourably to other methods for plastid transcript profiling.

This platform was used to quantify the changes in an unbiased group containing most chloroplast transcripts over the diurnal cycle in *C. reinhardtii*. Strikingly, this analysis revealed that transcripts behave differently depending on the function of their gene product. Unbiased clustering into three groups reliably separated photosynthesis-related transcripts, ribosome-related transcripts and RNA polymerase (PEP) transcripts.

Even more strikingly, these groups of transcripts fluctuate in a temporally ordered manner. PEP transcripts peak first at the end of the night, ribosome-related transcripts peak early in the day, and photosynthesis transcripts attain high levels at the end of the day. This agrees excellently with the hypothetical demands of a cell “waking up” from the night. Assuming that protein complexes decline during the night (which is likely due to the fast growth rate of *C. reinhardtii*, causing “dilution by division”), the cells will have to build up all complexes again for the day. Hence, remaining RNA polymerase can first transcribe its own genes, which can then be translated by remaining ribosomes in the chloroplast. In this way, a feed-forward loop creates more RNA polymerase. After a temporal delay, ribosome transcripts begin to increase, which are in turn translated by remaining ribosomes, creating another feed-forward loop on ribosome content. Finally, after a longer temporal delay, photosynthesis transcripts start to increase, which can then be translated by the large pool of ribosomes already built up.

Although substantial regulation of chloroplast gene expression also occurs at the translational level [30], the observed patterns in transcript levels are unlikely to be insignificant. It is also interesting in this respect that the window of peak transcript levels corresponds to the window of increased supercoiling of the chloroplast DNA [19]. Furthermore, our finding that a similar temporal order of gene expression over the diurnal cycle exists in the nucleus illustrates the tight co-regulation of plastid and nuclear gene expression through anterograde and retrograde signalling pathways [31], [32], [33]. In fact, our set of nucleus-encoded genes for chloroplast proteins included several translation factors (Dataset S1), whose expression also cycled diurnally (Figures 3 and 4), raising the possibility that translational regulation reinforces the changes occurring at the transcriptional level. Finally, it is also important to realize that the changes in transcript accumulation measured by microarray hybridization or qRT-PCR represent the result of transcriptional regulation and regulation at the level of RNA turnover. It is noteworthy in this respect that at least some chloroplast genes are also diurnally regulated at the level of mRNA degradation [34], [35], [36]. Consistent with this, several known (nucleus-encoded) proteins that determine the stability of specific chloroplast mRNAs were included in our analyses (Figure 3; Dataset S1) and showed diurnal rhythmicity.

In summary, our data reported here uncover a striking internal logic in the diurnal gene expression program of the model alga *C. reinhardtii*. The peak in the expression of genes related to transcription is followed by the peak in the expression of genes related to translation which in turn is followed by the peak in the expression of genes involved in photosynthesis. It will be interesting to see whether this pattern is conserved also in seed plants. Also, the identification of the *trans*-factors that confer this intriguing pattern of diurnal regulation represents an attractive challenge. In combination with the *cis*-elements they bind, these factors could provide useful tools for controlling gene and transgene expression in both the nuclear and the chloroplast genome.

## Materials and Methods

### Algal strains and growth conditions

*Chlamydomonas reinhardtii* strains CC-124 and SAG73.72 (equivalent to CC-3348 in the Chlamydomonas Resource Center; www.chlamycollection.org) were grown in bioreactors which allow the growth of 5 L of continuous culture in highly controlled conditions [22]. The reactors were bubbled with 200 cm^3 ^min^−1^ of filtered 5% CO_2_ in air and were stirred at 50 rpm with three impellers. Light was supplied from two custom-made light-emitting diode half-shells, and the light was focused with individual lenses to the center of the bioreactor, to minimize light gradients within the bioreactor. Cell density was monitored with a turbidimeter, and when the threshold of 0.4 or 0.5 was exceeded, sterile medium was pumped into the bioreactor to dilute the culture. The addition of medium was sensed as an increase in weight of the bioreactor, and a corresponding volume of culture was removed to maintain a steady volume. When the pH of the culture dropped below 6.95, or above 7.05, sterile 1 M KOH or 1 M HCl was slowly pumped into the bioreactor until pH 7.00 (+/−0.05) was attained. Temperature was maintained at 24.0°C by cooled water circulating within an outer mantle. The dissolved oxygen concentration was also measured. All of these parameters were logged in the BioPAT MFCS/win software (Sartorius; http://www.sartorius.com/en/products/process-control-tools-software/biopat-mfcs/), which also controlled all parameters of the bioreactor, including light in conjunction with a second software program, Quattro Color (ProBioData GmbH, Berlin, Germany).

Cells were grown in H_5_P media (Tris-minimal medium with 5 mM HEPES in place of 20 mM Tris; [37]). Cells were precultured in H_5_AP (Tris-acetate medium with 5 mM HEPES in place of 20 mM Tris) in continuous light, before bioreactor inoculation. Cultures were grown in constant light until they reached their target optical density, then they were allowed to grow for at least an additional two days in constant light. The cells were then exposed to three days of 12 h light/12 h dark cycles before samples were taken on the fourth day of diurnal conditions. Samples were centrifuged at 4000×*g* for 4 min at 4°C, and the cell pellet was frozen in liquid nitrogen until use.

### RNA extraction

RNA was extracted from 3–5×10^7^ cells with the Trifast reagent according to the manufacturer’s instructions (Peqlab, Erlangen, Germany). Contaminating DNA was removed by treatment with the TURBO DNA-free kit (Invitrogen, Paisley, UK). RNA quantity was estimated by measuring the absorbance of the RNA solution at 260 nm. RNA quality was determined by denaturing electrophoresis in formaldehyde-containing agarose gels using standard protocols.

### Primer design for qRT-PCR

Primers for qRT-PCR were designed with the online software QuantPrime (www.quantprime.de) after the chloroplast and mitochondrial genomes were added to a custom database. Additional primers were designed with Primer3 (http://primer3.ut.ee/). All primers were tested for acceptable melting curves and product sizes. Primer pairs which failed these tests were discarded and redesigned. After testing and redesign, primers for 68 chloroplast, 21 mitochondrial, and 51 nuclear transcripts were retained. These primers are listed in Table S1 in File S1.

### qRT-PCR experiments

cDNA was synthesized from 2 µg of total cellular RNA with the SuperScript III First-Strand Synthesis System for RT-PCR (Invitrogen) according to the manufacturer’s instructions. Each 5 µL qPCR reaction contained 2.5 µL Power Sybr Green PCR Master Mix (Applied Biosystems), 0.5 µL of diluted cDNA (corresponding to cDNA obtained from approximately 5 ng total RNA), and 2 µL of 5 pmol µL^−1^ forward and reverse primers). Reactions were conducted in opaque 384 well plates in a 7900HT Fast Real-Time PCR System Instrument (Applied Biosystems).

### Northern blotting and mRNA detection

Denaturing electrophoresis in formaldehyde-containing agarose gels was performed according to standard protocols. Electrophoretically separated RNAs were blotted onto nylon membranes (GE Healthcare) by capillary transfer. RNA was immobilized on the nylon membrane by UV crosslinking. Specific signals were detected with probes generated by PCR (Table S2 in File S1), purified from excised gel slices and labelled with ^32^P dCTP using the Megaprime DNA Labeling System (GE Healthcare). Membranes were stripped by washing several times with boiling 1% SDS.

### qRT-PCR data analysis

CT values were normalized to the geometric average of three housekeeping genes (*CBLP*, *BTUB* and *RBCS2*) to obtain ΔCT values. ΔCT values were mean centered per gene to the average across all time points.

k-means clustering was performed with the Pearson correlation metric using the Multiexperiment Viewer software package [38], after Figure of Merit analysis was performed to determine that 3 was a suitable value of k.

To classify transcript profiles to a sine curve, each individual transcript profile was compared to twelve sine curves (period 24 h, amplitude 0.5, phase offset by 2 h) using the Pearson correlation metric. The best match was used to classify the time at which that particular transcript peaks.

## Supporting Information

File S1 Contains the following files: **Table S1.** List of primers for qPCR used in this study. **Table S2.** List of PCR primers used to generate hybridization probes. **Figure S1.** Average expression levels of plastid transcripts across multiple experiments. The average expression level for each transcript was calculated for each experiment. The expression levels determine by qRT-PCR are shown on the left, and were used to sort the heatmap. This dataset is compared to two microarray experiments [22], [24] and one NanoString experiment [25]. Expression levels are all in log2 and are mean centered. High expression is indicated by red, low expression by green, and grey boxes indicate missing data. All experiments were performed with light-grown algal cultures, but the growth conditions were likely not identical in all details (e.g., light intensity, spectral quality, cell density, media composition), thus potentially explaining part of the variation.
(PDF)

Dataset S1 List of genes, PCR primers, gene functions, gene classifications, and associations with clusters.
(XLSX)
