# Supplementary material for: The Diurnal Logic of the Expression of the Chloroplast Genome in Chlamydomonas reinhardtii
Source: PLoS One. 2014 Oct 1;9(10):e108760. doi: 10.1371/journal.pone.0108760 (PMC4182738; doi:10.1371/journal.pone.0108760)
Supplement: File S1 — Contains the following files: Table S1. List of primers for qPCR used in this study. Table S2. List of PCR primers used to generate hybridization probes. Figure S1. Average expression levels of plastid transcripts across multiple experiments. The average expression level for each transcript was calculated for each experiment. The expression levels determine by qRT-PCR are shown on the left, and were used to sort the heatmap. This dataset is compared to two microarray experiments [22], [24] and one NanoString experiment [25]. Expression levels are all in log2 and are mean centered. High expression is indicated by red, low expression by green, and grey boxes indicate missing data. All experiments were performed with light-grown algal cultures, but the growth conditions were likely not identical in all details (e.g., light intensity, spectral quality, cell density, media composition), thus potentially explaining part of the variation. (PDF) [file pone.0108760.s001.pdf]

Supporting Information

Table S1. List of primers for qPCR used in this study.

**Gene**	**Forward primer (5'→ 3')**	**Reverse primer (5'→ 3')**
atpA	GGCTAACGCTAACGAACCAG	TCACGGTAAGCTTGTGCTTG
atpB	TTTACCACCACGACGGTATG	ACCGTACAGCTCCTGCTTTC
ATPC	AGCAGTACAACATCGTCAAGTCC	TCCGACTCCTGAGCAATGAACG
atpE	TGCTTATCCGTGGTGGTCAAGC	CAGCAGAAACGGCTTCGTTAGC
atpF	GCCGCACGTACGCAATTAGAAAC	ACCTTCTTCACGGATTTCTTGTGC
atpH	TGGTCCTGGTATGGGTCAAG	CCACGGATTTTACCTTCAGC
atpI	TAAGCACCCGCTAAAGTTGC	TGAACTTGTTGTTGGCGTTC
BTUB	CCCCCGCCTGCACTTCTTC	GTCGGCGGCGCACATCAT
CBLP	GCCACACCGAGTGGGTGTCGTGCG	CCTTGCCGCCCGAGGCGCACAGCG
ccsA	CCATTCGTTCCGTTAGCTTC	TATCCTGACGGGTTTCAAGC
cemA	GGGATCGCGAAGGTGAAGAAAC	TCGCAAGTTTGCACCAATAACACG
CHLAMY1 C3	AAGCTGTTTGTCGGCCAGATCC	CGTTGCCCTTGTTGAGTACGTG
chlB	TGCGGATTGGTTTAGAGAGC	CGTGACGTTCCATTTGTGTC
chlL	TGTTGTTTGTGGTGGCTTTGCTG	CCCGCTAAACGCAATGGATGTG
chlN	TGTTAGATGGTGCCACAACG	ACAGCTAATCGGGCAAAAAG
clpP	TCCACAGATTGACGTTTTGC	GGCACCGCAAAAACAAAAG
CLPP4	TGGATATTGAGATCCAGGCCAAGG	ATGATGCGGTTCAGGTTGGC
cob	CGCAAGATCATACCAGAAGG	TTCTTGGAACGGTGGTTCTC
cox1	CGCTATGGGTGCCATTAGTTTGC	AGGCCGACGGTAAACATGTGATG
CPH1	AAGACAGCCGTTGTGTGGTTCC	GGTATCACATTGGGTGCTGCTG
CRY	AAGCCACACACTGTATGACACG	TGGTGAACGACTGCATGGTGAG
EFG1	GTCTGCAGTCCTCTTTCAAGGC	TCCAGAGTCTTCTCACGACGAG
EFG3	AGCTGGTGATTGTGGAGCTACTGG	TGTGCAGCATGGACTTGTAGCC
EFG7	TTGCCTCATCGGATTCAAGAGC	ATTGCAGGAACACGCGGTTTAG
EFP	TCTTCGTTCGACCCAGGTGTTC	TCAGACCGTTCTTGAAGTCGTTCG
EFTS	TTCGGCGCTTTCGTGAACTTCG	AGACGCCAGCTTGGAAATGTGC
Factin	CTGACTCTGCGCTACCCCATT	CCTCAGTCAGCAGCACGGG
IF1	TTGACTGCACGCGGATGTATGG	ACGTCAGATGTGACACGGAGAG
IF2	AGAACACGGTCAAGGTGGTTGC	AATGTCCTCCTCCGTGACGAACTC
IF3	ATGCGCATGCGTTATGACGACCTC	AAACACCTCCTTCACCGTGATGCC
LHCA1	TGTGAGCAACGTGAGAGCAAGG	ACCACCACAAGACCAACCAACAC
MBB1	TGCAGCAGAAGCGCTATGATGAAG	TGGGTTGACGTTGCCTGTATTGG
MBD1	ACAAGGCGCGATTGCTGTTC	CCTCAAAGCGAGCGTACATGTTG
MCA1	AGCAGTTCGTGTGGCTGTGATG	ACTCAATCGAGTCCCTCTGTGC
MCD1	AACATGGTCCCATGATGAGCAG	CATGAGGTGGATGAGCAAGCAAG
MRL1	CAGGAGCTGAACAAACCCATGC	ATGAAGGACGAGTAGTGCCACCTC
mtNEP	AGGATGGCTGTGTGTGTGTGTG	ACCCTGCAAACAAACCCTGTTCTC
nad1	TCATGCGCCTAGGTTGGAAAGC	AGCATGCAAGGCGAAGAAAGCC
nad2	GCTTGCCACCATTTGCAGGTTTC	CGCTCATAGCATGCCAGAAGATCC
nad4	TGCTTACGTGTTCTGCGTATGCC	ACACGGTTGAAAGCCCAGAAGC

nad5	CAGCCATAAAAGCGGTCAAG	CCTAATTCACGCCGCTACTC
nad6	TGCTACTAGAGATCCCAGCCACTG	CCCAAAGTACTCCAACCACGACTG
NMD	TGGAAGAAGACCGTCAAGGTTGCC	CCGGAAGCAAGCATGAATAGCAGG
orf112	AAAGTTCCTCCACTGCCTCCTTC	AATACTTCGCAACTGCCACTGAC
orf140	CAAGGCTTTGGCTTGTTAGGAAAC	GCTTCTTGGATTTCCAGCGCAT
orf1995	CAACTAAAGCTGCAAGTGGACCTC	ACGGGCTTCAGCTGGATTAGTTTG
orf271	AAAACCGACAAGAAAGGAACC	GCAAAAGGTTGGGAAACAAG
orf2971	TCTGTACCTGCTGGTGCTAGTG	ACGTGAACTCGATTGTGCTACTGG
orf50	ATCGAAGGACGTCCCAAAGAGG	TGTGGTCTGGCTACGAACTGTG
orf59	TGTGGGATGACCGGTTAGGTACTC	AAACTGCCATACCGACCAAGCG
PDI2	CGGCTTCCCTACCATCAAGTTC	TGTAGTCCTGCATGTTGTCCTTGG
petA	TTTACGCGCAGCAACATTAG	TGCTTTTTGCGCTAAGTGAC
petB	TGTTCCTGATGCAATCCCAGGTG	AGTTGCTTGACCAACACCAACAC
PETC	CGTAAATCCGCTCCTGCTACTG	TGGCTGAGAGAGACAATCAACACG
petD	ACTTACGGTGAACCTGCTTG	GTTTGCTGGCTCACCCATAG
petG	GTTTTAGGCTTAGTTCCAGTAACG	TGTAGCTAAATCACCACGCAGA
PHT1	TTGGTCACAACTGCCGCTTC	TGATGGCGTCCCTGATCTTCTG
PRPL6	AGAAGCGCATGGAGATGGTTGG	ACGTTCAGGGTCAGATCCTTGC
psaA	TGGGGTACGGTTACAGCTTC	TGTGCCCATAAGAAGTCACG
psaB	TCCAGAATCACGTGGTCAAC	TGAGCAGTACCGAAAACGTG
psaC	GGTTCCATGGGATGGTTGTAAAGC	ACACAGTCTTCAGTGCGTGGAG
PSAE	ACGACGAACAACTACGCTCTGG	CGCGAAGCCAGCCATTTACTTG
psaJ	CAAGTGGATCAGGGAAATAACG	TCAACAGCACCTGTAATTGCTA
psbA	CATCCTTATGCACCCATTCC	TGACCGAAACGGTAACCTTC
psbB	AGCAGTATTCTGGGCAGCTTTCG	AGTAGCTGCTGAACCGTACCAC
psbC	AATTGTTTTCTGGGCAGGTG	CCTAAAGTTGCGATGTGTGG
psbD	GCCTGTGTAGGGTTGAATGC	TGCTGGTGTTTTAGGTGCTG
psbE	TTCATTTGGACGTGGAGTACC	TGGCTGGTAAACCAGTAGAGC
psbH	TGGCAACAGGAACTTCTAAAGCTA	CCTGCTTCTGAGTTAAGTGGACG
psbK	TGAAGCATACGCACCATTTGCAC	CGGAAACTAACAGCTGCTTGCC
PSBO	CCGCTGCTCTCAGTCAATAACAGG	ACACGCCTCATGACTAATCTGTGC
psbZ	TCCTTCAAGTTGCCCTACTTGCTT	GTCAGTCCAACCATTAGGAGTTGC
PSRP3	AGACAACACAGCTGGTAGTGGAG	ATACGCGCCATACCCATGACAG
RAA1	ACAGGAGTGGATGCTTGCGTTG	ACAGGCCCTCTGTGTCAAACTG
RAA2	CATGCGCACATTGGACATGGAG	TGATCGCGTCCATACCAAACCG
RAA3	AACCCGCTTGTAGAGCTGAAGTTG	ATCCAGCTGCGGAAACAGTGAC
RAA4	TGCTGATTTGGAGGAGCAGAAAC	CGAATAATGTGCAGGAACAACTGG
rbcL	TCGTGAAGGTGGCGACGTAATTC	CACATGCAGCAGCAAGTTCTGG
RBCS2	ATACTGCTCTCAAGTGCTGAAGCG	AAAGACTGATCAGCACGAAACGG
RBP40	TGACAACCGCAACAACAAGCAGAC	ACAACAGCTCGTTGAGCCACTC
RF1	TGGGATCAGAGGATCTCAACGG	TGTACTGCCTGTACGACACAGC
RF2	GCCGGGTGATTCATGTTTGTGC	AGCGCATTTACCCTTGCATTTACC
RF3	AAGGTGACGCCGGTGTTCTTTG	TGAAGGTCTGCAGGAACAGCTC
ROC81	ATTCCAGCCAGGAGCTCTTCTTC	ACTTGTTGACCTTCTCCACATCGC
rpl14	TCTTGGTGGCAGTTATCGTG	CCATTTTCACGACGGATACC
rpl16	ACCACACCGTGGTCATTTAAGAGG	CGTCCGGCTTCAATTTGACGTG
rpl2	CAGTACGTGGTTCGGTGATG	ACCCCATGGTGTAACTGGAC
rpl20	CACATACGACGGAAATCACG	TTTAGAGGCGCTGCTTCAAC
rpl23	ACGCATATTCCACCACGTCAAA	ACGTGGACGATAACCTTGTGCT
rpl5	AGACAACAAATGCCCGTAGGTG	AAATCGCGTACACGCGGTAATG

rpoA	GCCCACGTGTAATTAAAGCCGC	ACACATTGTAAACCTGCTGGTAGT
rpoB-1	TCAGCCACGTTAGCCATTCGAG	GGGTTTCAACGGGACAAATACGAC
rpoB-2	CCCACGGCATATAAGCTACG	ACCAGCGGTTAAAGAAGGTG
rpoC1a	AACCAAACATGGGTTGTTGG	AGCGGAAAGTCGTCAAACAC
rpoC1b	CAAATATTGGCGGAATCGAC	GTAGTGCCCGAAAAAGATGC
rpoC2	TCAGGCAGGGAAAGTGCTAC	ATGGCCTTTGTTCCAGATTG
RPOD	CATGGATGTCGAGAGCCTGAAG	ACACCGTCATGACCAGCTTGTAG
rps11	TTTAGTAACAGGCCCGGGTCAAGG	TCGGTGGACGACAACCATTGTG
rps12	TGCACTTCGTAAAGTTGCTAGGG	GGCCAACACCTGGAATATAAGCTG
rps14	CAGCAGTGCGTTTACACAACCG	GCCTTTAGGACGGCCTGTAATCA
rps18	CAAACGGTAACAAGCCCATT	GAAGACAAACACGTTTAACAGC
rps19	GGTGTTTATAATGGGCGTGA	CATGGCCACGATATGTACG
rps2	ATTTGACGCAGACTTGAACG	TGGTTCAGCAGGCAATTTAAC
rps3	TCAAGCAATGGCCAATGAATCACG	CTTTACGAGCAGTCGCAATTGTTG
rps4	AATATGGCACCTACAATTCCAGCA	TGTCCATGACTAATAAGTTGGCGT
rps7	TCGCGCTCGAGCTAACTCATTAC	TGGTTGACCTGAGCGCTTATCG
rps8	CCAGGTTTACGCATTTATTCA	CACGTAGACGAGCTTCACGA
rps9	TAGCTAAAGCTTCGCCTTCC	TTAATGGGCCAAACTGAAGC
RRF1	TGAAGAAGGTGGACAAGGTTGAGC	ACGTGTCAGTCAGCTTCTGGATG
rrn5	TCAGTTGGACCCACACCAATCC	ACGGCGACCCGTAAAGTTCTTC
rrn7	AGACGAAGAAGGGCGCAGATAC	GGATCAAAGCTTGTTGCCAGCTC
rrnL	AAACGTCGTGAGACAGTTTGGTC	AGCTACCCAGCGTTTCCCATTG
rrnL1	AAGGGATTGCTGACTGATAGTGC	ATGCGCGAATGCTCACGTATTG
rrnL2b	GCGTGCAACAACACACTAAAGGG	TGTGAGAGTGTGGGTTGAACCAG
rrnL3a	ACTCAATTTCGGTGCTCGGTGA	GGGTCCCAGTACTAAGCCAGTAA
rrnL3b	AACCGATCGATCTAGCGACGTG	TCTTGCAACAGACACGGGTTCG
rrnL4	ATACCGGTAGCGCAAGCGAATG	TGACCTTACCGCCGATTGTCTG
rrnL5	AAGGTAGAGTCTGCGTTGAACACC	TTCCAAGCACAGTGGCTGCTTC
rrnL6	ACGTTCTGGAACAATTACGCTGA	GGGCTTACGGCCATGTTATCCA
rrnL7	ACGTTTAATTGGCGTCCTTG	AGCTGCATAGGGTCTCTTCG
rrnL8	CGCAAGGGTTGGATTGTTCGTC	AGAAACCGACCTGTCTCACGAC
rrnS	TGGAGGAAGGTGAGGATGAC	TTCATGTAGGCGAGTTGCAG
rrnS1	GTTTGGTGCTGGCTCAGCTTTC	ATTACGCACTCGTGGCCTTAATC
rrnS2	CCCATGGCTTATCCTTAGCCTGTC	ATCCGCCGTTCAAGCGATTGTC
rrnS3	ATTCCGGATGGAAAGCCGAAGG	CGCTACCCAAACTTACGCGTTG
rrnS4	ACGGTCGAGCAAGTCATCATGG	TGGTATCCAACTTGTGGGTGTCC
rtl	TACGCCGACAGTACATCCAG	TACCAAAACCAGGACGGAAG
TAB2	CCGCTGCTTCACTGTCATGTCTTG	TCCTGCTTGTACACCGACTCTAGC
TAB3	CACAGATGTACTCGGTGTAGCG	ACCGAGGTTCTTCAGTGATGGC
TBA1	GTCATGAAGAAGGCTTTCGACGAG	AGGAGTCCAGAGACAGAGTCTTGC
TBC2	TGGCACGTGAATCCAGCATTTG	TCGCTCTCGCAGTTACCATCTTC
TCA1	GCGTGTTCACGCATTTGTGTTTC	TGACGATCCCAAACCGACCAAG
TDA1	TGCGTAGATGCCTGCTTGACAAC	CGCGCAGAAACTTCCTTGCTTC
TPR6	AGCTGAACAAGGTCACCAAGGC	TGGCAGCAGTCTGGTACACATC
tscA	TCCAACTTCAGCTAGGTTTGATGT	ACGAAAGACCACAATAAGCCCT
tufA	TACATCCCAACTCCACAACG	AATGCACCACGTTCTACACG
VIPP1	ACACGTGCTGCGGACTGTAAAC	TGTGCAGTGGCATTAGCGTTCAG
ycf3	AACTGCGATCGTAAGCATCC	ATGTCAGCACAAGCTGAAGG
ycf4	AAAACCACCACCAACATTCC	TGTTCCGTTTTTCCCACAAG

Table S2. List of PCR primers used to generate hybridization probes.

Transcript	Forward primer (5’→ 3’)	Reverse primer (5’→ 3’)
rpoA	CGTTGAGTAAGTAACGTGCGT	GTGTAAATGGCCCACGTGTA
rps2	TGCATCATCATTAGCTGGAA	ACAAATTGCAGCCCAAAAAC
rps3	ATCGTGGTGCTACTCAACCA	CGTGATTCATTGGCCATTGC
psbB	CACAATCTTGGGGTGGTTGG	GATGCGTGACCTAACCAACC
tufA	GACGGTCCTATGCCACAAAC	TTGGGATGTAGCTGTCAACG
psaB	CACAACGACGTAATGCAAGC	GCAATAGCGTGGTGAACAAG

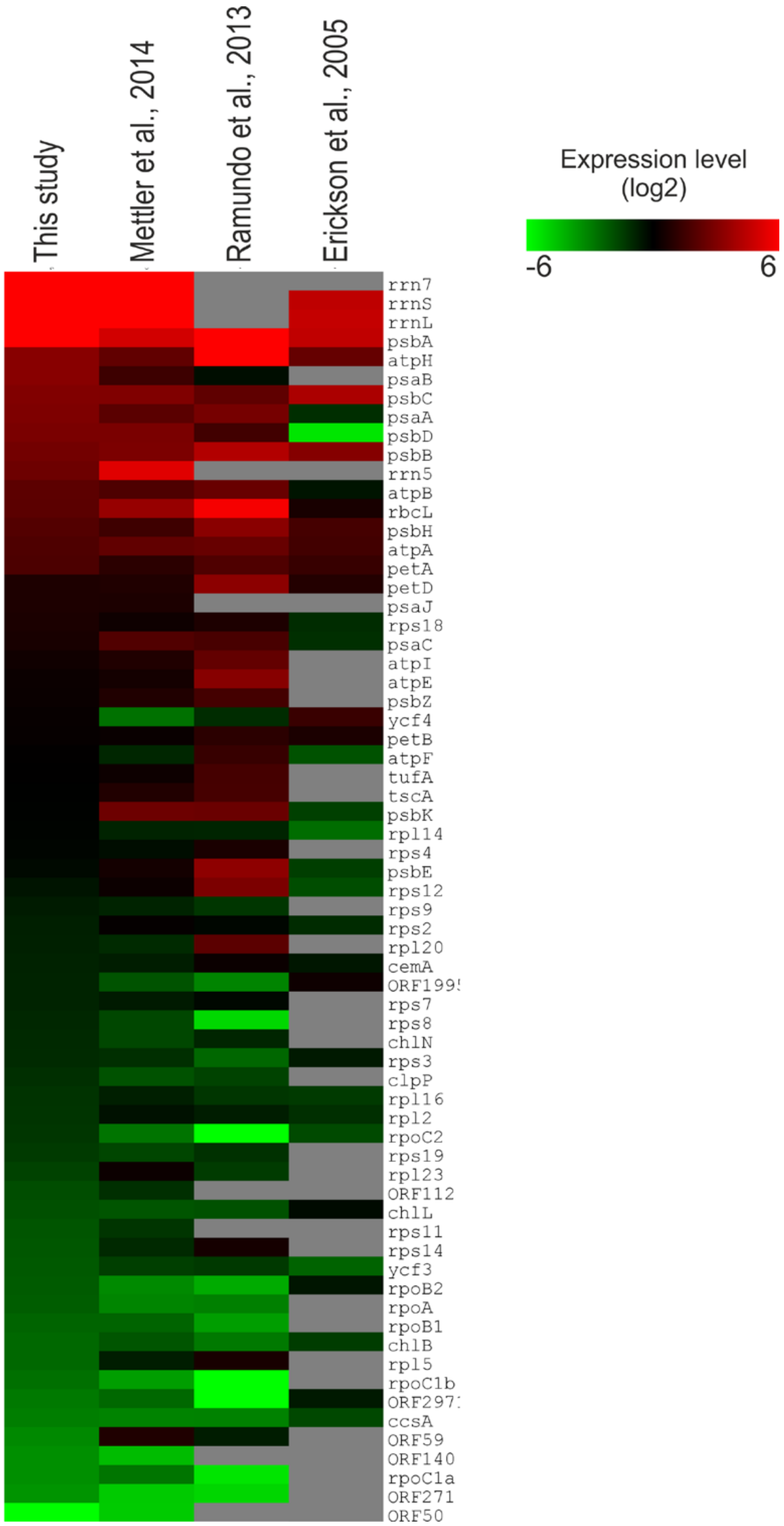
This study
Mettler et al., 2014
Ramundo et al., 2013
Erickson et al., 2005
Expression level
(log2)
-6
6
rrn7
rrnS
rrnL
psbA
atpH
psaB
psbC
psaA
psbD
psbB
rrn5
atpB
rbcL
psbH
atpA
petA
petD
psaJ
rps18
psaC
atpI
atpE
psbZ
ycf4
petB
atpF
tufA
tscA
psbK
rpl14
rps4
psbE
rps12
rps9
rps2
rpl20
cemA
rps7
rps8
chlN
rps3
clpP
rpl16
rpl2
rpoC2
rps19
rpl23
ORF112
chlL
rps11
rps14
ycf3
rpoB2
rpoA
rpoB1
chlB
rpl5
rpoC1b
ccsA
ORF59
ORF140
rpoC1a
ORF271
ORF50

Figure S1 Average expression levels of plastid transcripts across multiple experiments. The average expression level for each transcript was calculated for each experiment. The expression levels determine by qRT-PCR are shown on the left, and were used to sort the heatmap. This dataset is compared to two microarray experiments [22], [24] and one NanoString experiment [25]. Expression levels are all in log2 and are mean centered. High expression is indicated by red, low expression by green, and grey boxes indicate missing data. All experiments were performed with light-grown algal cultures, but the growth conditions were likely not identical in all details (e.g., light intensity, spectral quality, cell density, media composition), thus potentially explaining part of the variation.
